# Preparation and Properties of Hydrophobically Modified Nano-SiO_2_ with Hexadecyltrimethoxysilane

**DOI:** 10.1021/acsomega.1c00381

**Published:** 2021-03-31

**Authors:** Bingbing Xu, Qiuhui Zhang

**Affiliations:** MOE Key Laboratory of Wooden Material Science and Application, Beijing Forestry University, Beijing 100083, China

## Abstract

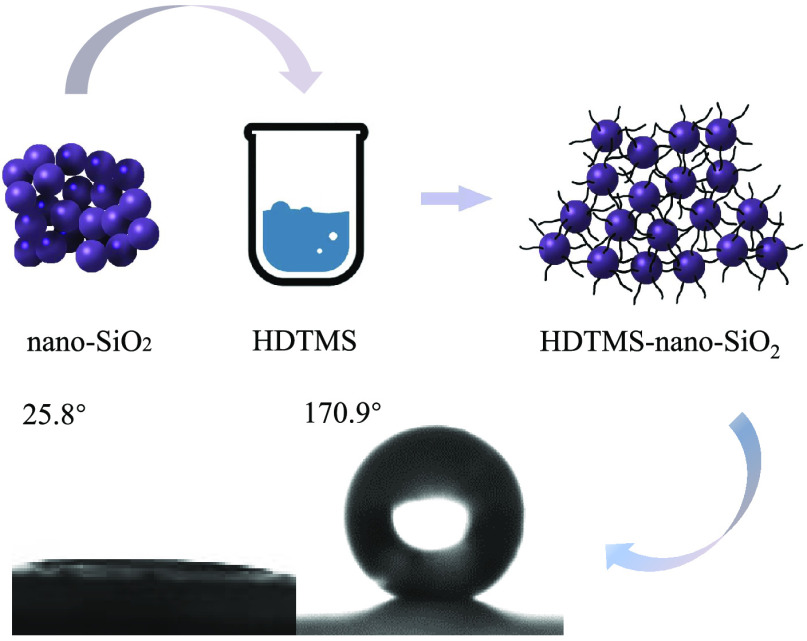

As a common inorganic silicon material, nano-SiO_2_ is extremely hydrophilic due to the presence of a large number of hydroxyl groups on its surface, which limits its application in some fields. In this research, hexadecyltrimethoxysilane (HDTMS) was used to modify nano-SiO_2_, and the results of water contact angle (WCA), Fourier transform infrared (FTIR), two-dimensional correlation spectroscopy (2D-COS), thermogravimetric (TG) analysis, and scanning electron microscopy (SEM) indicated that the hydrophobic long-chain alkyl of HDTMS was successfully grafted onto the surface of nano-SiO_2_. When the ratio of nano-SiO_2_ and HDTMS was 0.25:1, the WCA of nano-SiO_2_ modified with HDTMS (HDTMS-nano-SiO_2_) reached 170.9°, which was about 5.62 times higher than that before modification, and the superhydrophobic property was obtained. The novelty of this work lies in the modified nano-SiO_2_ with a WCA of over 170° and the analysis of the modification mechanism with the help of 2D-COS. This study can provide a reference for the hydrophobic modification of nano-SiO_2_ and its application field expansion.

## 1 Introduction

Nano-SiO_2_ is nontoxic and odorless with large specific surface area and light weight. Because of the excellent barrier, mechanical, optical, and flame retardant properties, it has attracted great attention in the fields of ceramics, paper making, plastics, metals, and photovoltaics.^1−6^ For instance, it can be added to polymer cells as a filler to slow down the battery’s capacity decay.^7^ Yu et al. prepared polylactic acid/thermoplastic polyurethane/ hydrophobic nano-SiO_2_ composites and improved the hydrophobic and mechanical properties simultaneously.^8^ Kumar et al. modified nano-SiO_2_ with polymethylmethacrylate to obtain both hydrophobic and corrosion-resistant coatings.^9^ Elkalla et al. synthesized polystyrene/hydrophobic nano-SiO_2_ composite particles by oil-in-water Pickering emulsion polymerization.^10^

It can be seen that the hydrophobic property is one of the important properties of nano-SiO_2_ being studied and applied. Nano-SiO_2_ is hydrophilic due to its large number of hydroxyl groups on the surface, so it is necessary to conduct hydrophobic modification research on it so as to expand its application field and deepen the research on the material surface.^11^ The key to preparing a superhydrophobic coating on the substrate surface is to construct the rough structure of binary micro-nano or modify it with low-surface-energy substances to reduce its surface energy.^12−15^ As a typical low-surface-energy substance, fluorocarbons have been commonly investigated in hydrophobic modification of such materials.^16−18^ Dou et al. modified nano-SiO_2_ with 1*H*,1*H*,2*H*,2*H*-perfluorodecyltrichoxysilane (PFDS), and the WCA reached 130.6°.^19^ Lin et al. successfully prepared a hydrophobic silica gel film with porosity over 90% by the sol–gel method using the hydrophobic fluorocarbon functional group–CF_3_ as a modified group, which can be used to absorb CO_2_.^20^ Nevertheless, fluorinated compounds are expensive and may accumulate and become toxic in organisms and the environment.^21,22^ Therefore, some hydrophobically modified substances without fluorine have caused extensive concern, most of which work by the reaction between the hydroxyl groups on the surface of nanoparticles and the hydrophobic groups of the modified substances.^23−27^ The silane coupling agent is one of the most commonly used modified substances with special properties, such as hydrolyzable chemical groups and organic functional groups. Its silanoxy groups are consequently reactive to inorganic substances, and its organic functional groups are reactive or compatible with organic substances.^28−30^ Kapridaki et al. prepared a titanium–SiO_2_–polydimethylsiloxane nanocomposite hydrophobic coating, which had excellent self-cleaning properties and could be used for cultural relic protection.^31^ Li et al. obtained a hydrophobic paper with a static contact angle of 163° and a rolling angle of 3° by spraying nano-SiO_2_ with octadecyltrichlorosilane (OTS).^32^ Tsuru et al. modified nano-SiO_2_ with methyltriethoxysilane (MTES) to obtain hydrophobic nano-SiO_2_ with a WCA of over 150°.^33^

Hexadecyltrimethoxysilane (HDTMS) is also a silane coupling agent, which has been frequently used in the hydrophobic modification of nano-SiO_2_. Xu et al. prepared HDTMS-modified nano-SiO_2_ via the sol–gel reaction under alkaline conditions, which was coated onto the cotton fabric sample. Then, the sample was endowed with hydrophobicity, the WCA of which was 152.1°.^34^ Sohrabi et al. modified nano-SiO_2_ with HDTMS and deposited it on glass. As a result, the glass had a WCA of 139.5° with 0.1% of the modified nano-SiO_2_.^35^ Xiong et al. synthesized a kind of functional nano-SiO_2_ with 2,4-dihydroxybenzophenone and HDTMS and then introduced it onto the surface of cotton fabric. The results indicated that the modified cotton fabric not only showed strong UV-resistant ability but also obtained the hydrophobic property with a WCA of 153°. Moreover, the modified cotton fabric could keep these properties after being abraded 300 times or laundered 20 times.^36^ Therefore, it can be seen that HDTMS has been successfully applied as a kind of hydrophobic modifier, which can make nano-SiO_2_ more hydrophobic. Besides, because of being widely used for cotton fabric, HDTMS-modified hydrophobic nano-SiO_2_ probably has potential in other materials and fields.

To study this subject, in this paper, we propose to utilize HDTMS as the modifier to modify the surface of nano-SiO_2_ and to explore the influence of different proportions of reactants on the hydrophobic property of nano-SiO_2_. WCA, FTIR, 2D-COS, TG, and SEM analysis were used to analyze the results. As a result, superhydrophobic HDTMS-nano-SiO_2_ was prepared successfully, the WCA of which reached 170.9°. In addition, the chemical mechanism of modification in this study was revealed. To our knowledge, hydrophobic nano-SiO_2_ modified by the silane coupling agent with a WCA of over 170° has not been reported. Moreover, the temperature sensitivity of chemical functional groups and the sequence of chemical changes were investigated with the help of 2D-COS, which has not been widely used in this field. The results of this study can provide a reference for the hydrophobic modification of nano-SiO_2_.

## 2 Results and Discussion

### 2.1 Hydrophobicity Analysis

**Figure 1:**
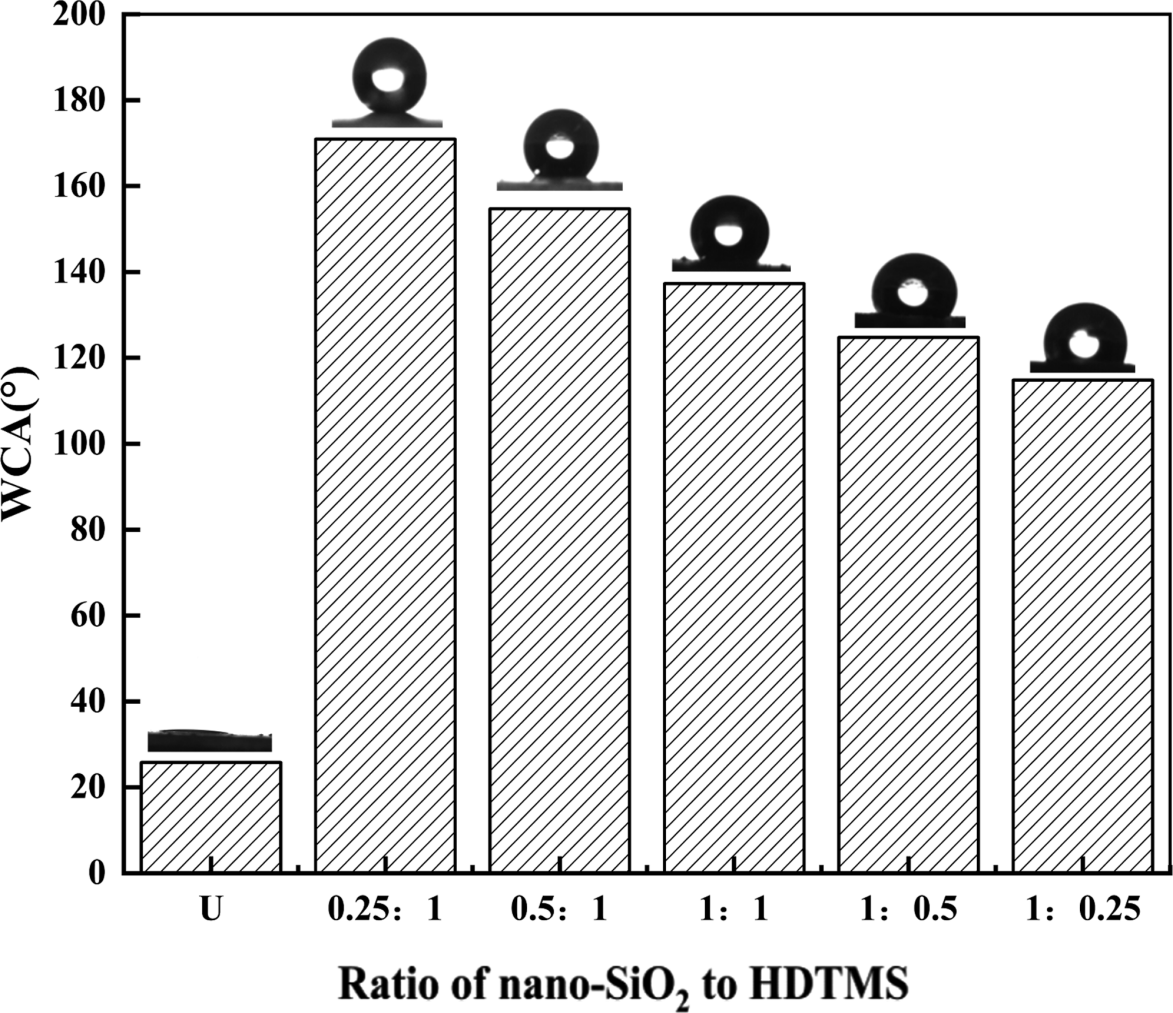
WCA values of unmodified nano-SiO_2_ (U) and HDTMS-nano-SiO_2_ when the ratios of nano-SiO_2_ to HDTMS were 0.25:1, 0.5:1, 1:1, 1:0.5, and 1:0.25.

The test results of the WCA value of HDTMS-nano-SiO_2_ obtained by the reaction of different proportions of nano-SiO_2_ and HDTMS are shown in Figure 1. Unmodified nano-SiO_2_ was so hydrophilic that water drops spread out quickly when hitting its surface, with a WCA of only 25.8°. When the ratios of nano-SiO_2_ to HDTMS were 0.25:1, 0.5:1, 1:1, 1:0.5, and 1:0.25, the WCA values of the modified nano-SiO_2_ were 170.9, 154.7, 137.3, 124.8, and 114.8°, respectively. This indicates that the hydrophobicity of nano-SiO_2_ modified by HDTMS was improved, and the hydrophobicity achieved the best effect when the ratio of them was 0.25:1. Compared with nano-SiO_2_ before modification, the WCA increased by about 5.62 times.

### 2.2 FTIR Analysis

**Figure 2:**
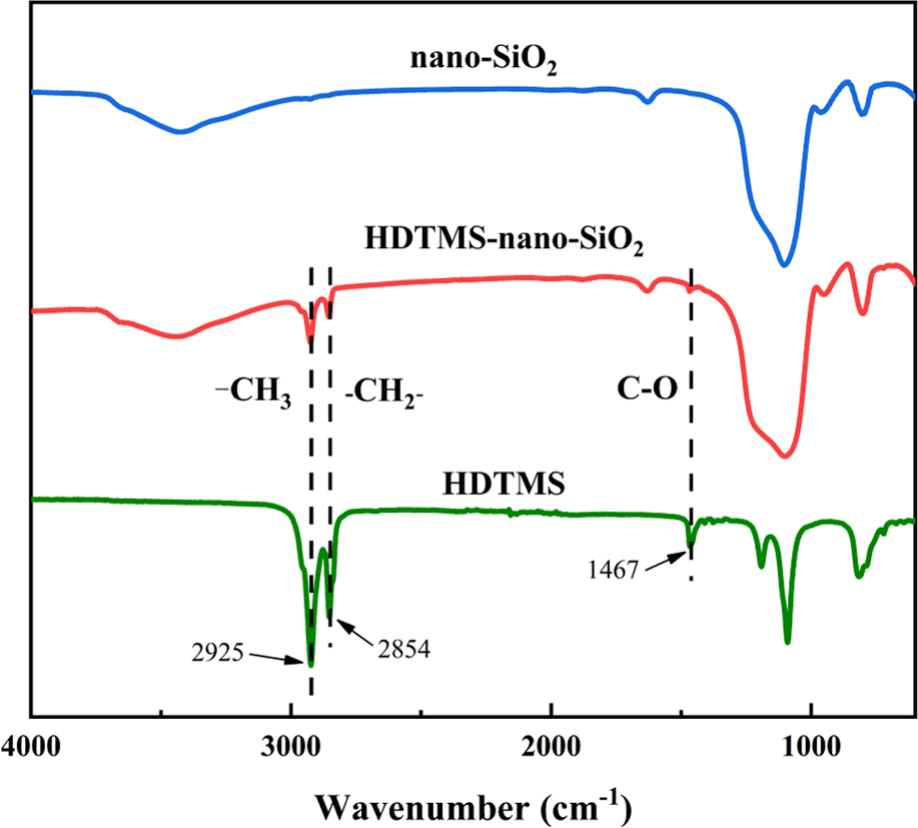
FTIR spectra of HDTMS, nano-SiO_2_, and HDTMS-nano-SiO_2_.

The change of functional groups of nano-SiO_2_ before and after modification was analyzed by FTIR, and the results are shown in Figure 2. HDTMS, nano-SiO_2_, and HDTMS-nano-SiO_2_ all had absorption peaks at 1100 and 800 cm^–1^, which were the antisymmetric and symmetric contraction vibration peaks of the Si–O–Si bond, respectively.^37,38^ At about 3440 and 950 cm^–1^, both HDTMS-nano-SiO_2_ and nano-SiO_2_ had absorption peaks. These peaks were assigned to the stretching vibration peaks of silicon hydroxyl groups on the surface of nano-SiO_2_ particles,^39^ and the absorption peaks of HDTMS-nano-SiO_2_ in these two places were slightly weaker than that of nano-SiO_2_, which proved that the surface of HDTMS-nano-SiO_2_ had been successfully modified and the hydroxyl groups were relatively reduced. Compared with the infrared spectra of the unmodified nano-SiO_2_, three new absorption peaks representing the stretching vibrations of −CH_3_, −CH_2_, and the C–O bond, respectively, appeared at about 2925, 2854, and 1467 cm^–1^, which indicated that the long-chain hydrophobic alkyl groups of HDTMS had been successfully grafted onto the surface of nano-SiO_2_.^11,40^

### 2.3 2D-COS Analysis

**Figure 3:**
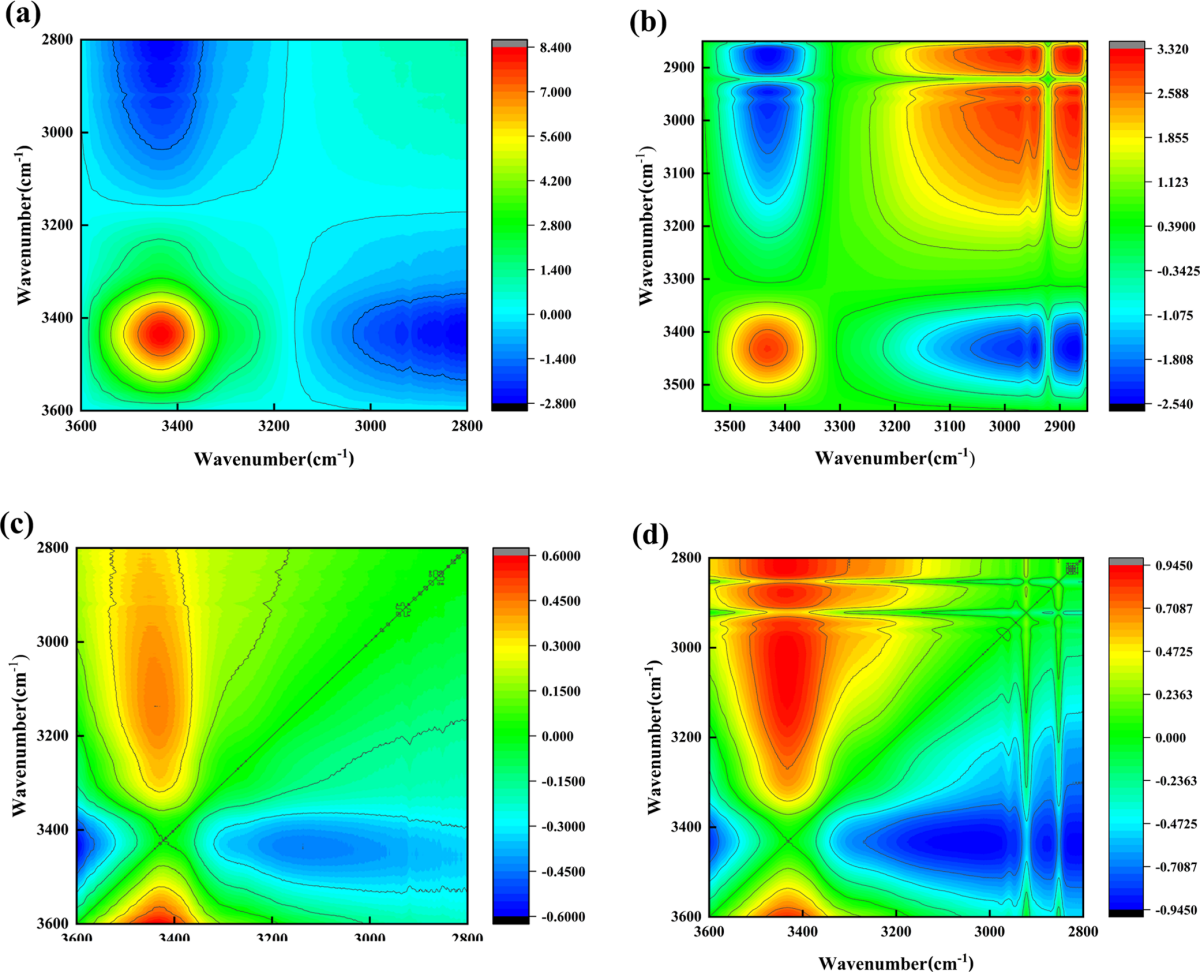
Synchronous graphs of nano-SiO_2_ (a) and HDTMS-nano-SiO_2_ (b) and the asynchronous graphs of nano-SiO_2_ (c) and HDTMS-nano-SiO_2_ (d).

2D-COS results are depicted in Figure 3. The synchronous graph of 2D-COS was used to indicate the degree of coordination between the two spectral intensity changes. The value of the automatic peak located on the diagonal is always positive.^41−46^ In the synchronous figure of HDTMS-nano-SiO_2_, three automatic peaks appeared at about 2925, 2854, and 3440 cm^–1^, which represented −CH_3_, −CH_2_–, and hydroxyl groups, respectively, on the surface of nano-SiO_2_. In the synchronous graph of this band, there was only an automatic peak at about 3440 cm^–1^, proving that the surface hydroxyl groups of nano-SiO_2_ were sensitive to temperature perturbation to a certain extent before and after modification. Besides, the existence of diagonal cross peaks on both sides shows that functional groups may exist in intramolecular or intermolecular interactions. The parsing rules are that two variables under perturbation of changes in the spectral peaks are positively related if the value is positive. On the contrary, these two variables under perturbation of changes in the spectral peaks are negatively related with a negative value.^41−46^

**Figure 4:**
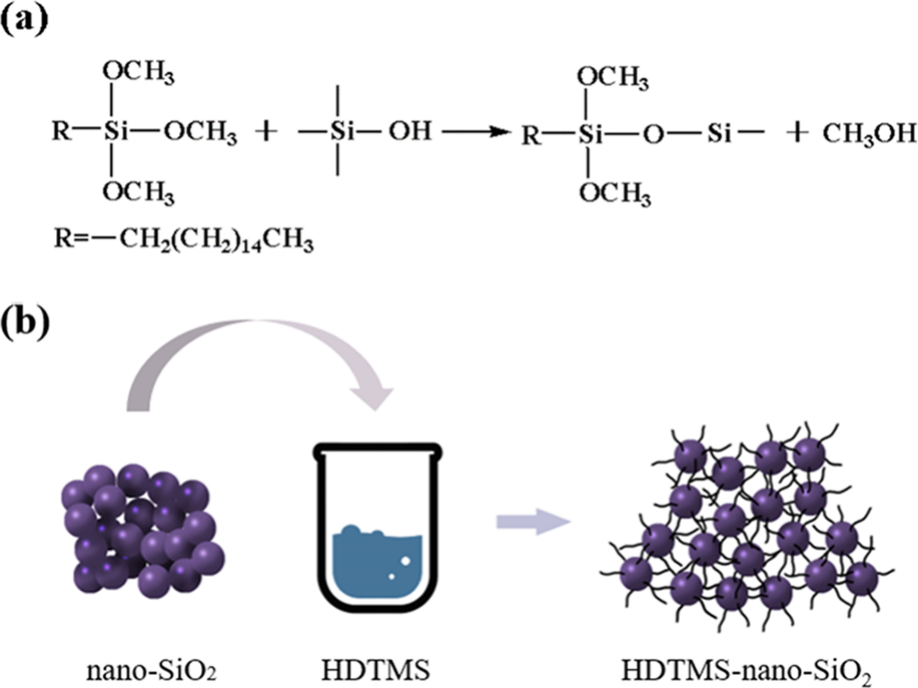
Reaction process (a) and the interaction of HDTMS and nano-SiO_2_ (b).

The asynchronous graph represents the degree of difference in the intensity changes of the two spectra. There are no automatic peaks and only cross peaks with positive or negative values in an asynchronous graph, which is antisymmetric about the diagonal. According to the rules of Noda, when synchronous correlation peak intensity Φ(ν_1_,ν_2_) and asynchronous correlation peak intensity Ψ(ν_1_,ν_2_) have the same sign, the change of the correlation peak of ν_1_ is earlier than that of ν_2_. Inversely, when synchronous correlation peak intensity Φ(ν_1_,ν_2_) and asynchronous correlation peak intensity Ψ(ν_1_,ν_2_) have different signs, the change of the correlation peak of ν_1_ is posterior to that of ν_2_. Therefore, the order of change of these groups with temperature perturbation can be acquired by combining their synchronous and asynchronous graphs.^41−46^ It could be found that the cross peaks (2925, 3854) cm^–1^ of HDTMS-nano-SiO_2_ had the same sign in synchronous and asynchronous graphs, hence the change of −CH_3_ at 2925 cm^–1^ was prior to that of −CH_2_– at 2854 cm^–1^. Because of the different signs in the synchronous and asynchronous graphs of the cross peaks (3440, 2854) cm^–1^, so the change of the hydroxyl groups at 3440 cm^–1^ is later than the change of −CH_2_– at 2854 cm^–1^. Overall, the sequence of changes of these three groups was −CH_3_, −CH_2_–, and hydroxyl groups. Combined with the results of FTIR analysis, it could be deduced that the reaction mechanism of hydrophobic modification was that HDTMS directly reacted with the hydroxyl groups of nano-SiO_2_, thus grafting its hydrophobic long-chain alkyl group onto the surface of nano-SiO_2_.^47^ The reaction process and the interaction of HDTMS and nano-SiO_2_ are presented in Figure 4.

### 2.4 TG Analysis

**Figure 5:**
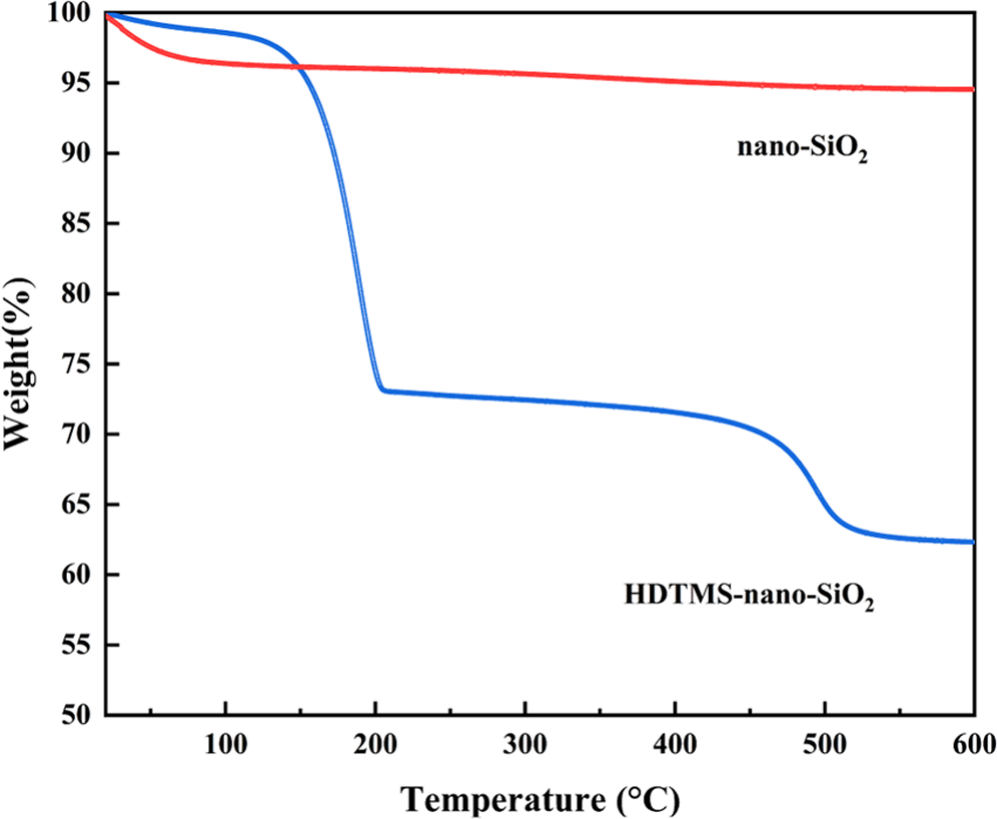
TG analysis of nano-SiO_2_ and HDTMS-nano-SiO_2_.

As exhibited in Figure 5, in the range of 20–150 °C, both nano-SiO_2_ and HDTMS-nano-SiO_2_ had a small amount of mass loss, and the mass loss of nano-SiO_2_ was more than that of HDTMS-nano-SiO_2_. Combined with FTIR and 2D-COS analysis, it could be inferred that the main component of mass loss was the hydroxyl groups on the surface of nano-SiO_2_. With the increase of temperature, the mass loss of nano-SiO_2_ was very slow, and the final mass remained about 95%. However, HDTMS-nano-SiO_2_ was decomposed for the second time in the range of 150–500 °C, resulting in a large mass loss of the sample with a final remaining mass of about 63%, which was mainly caused by the decomposition of long-chain alkyl on its surface. This also indicated that −CH_2_(CH_2_)_14_CH_3_ was efficaciously grafted onto the surface of HDTMS-nano-SiO_2_.

### 2.5 SEM Analysis

**Figure 6:**
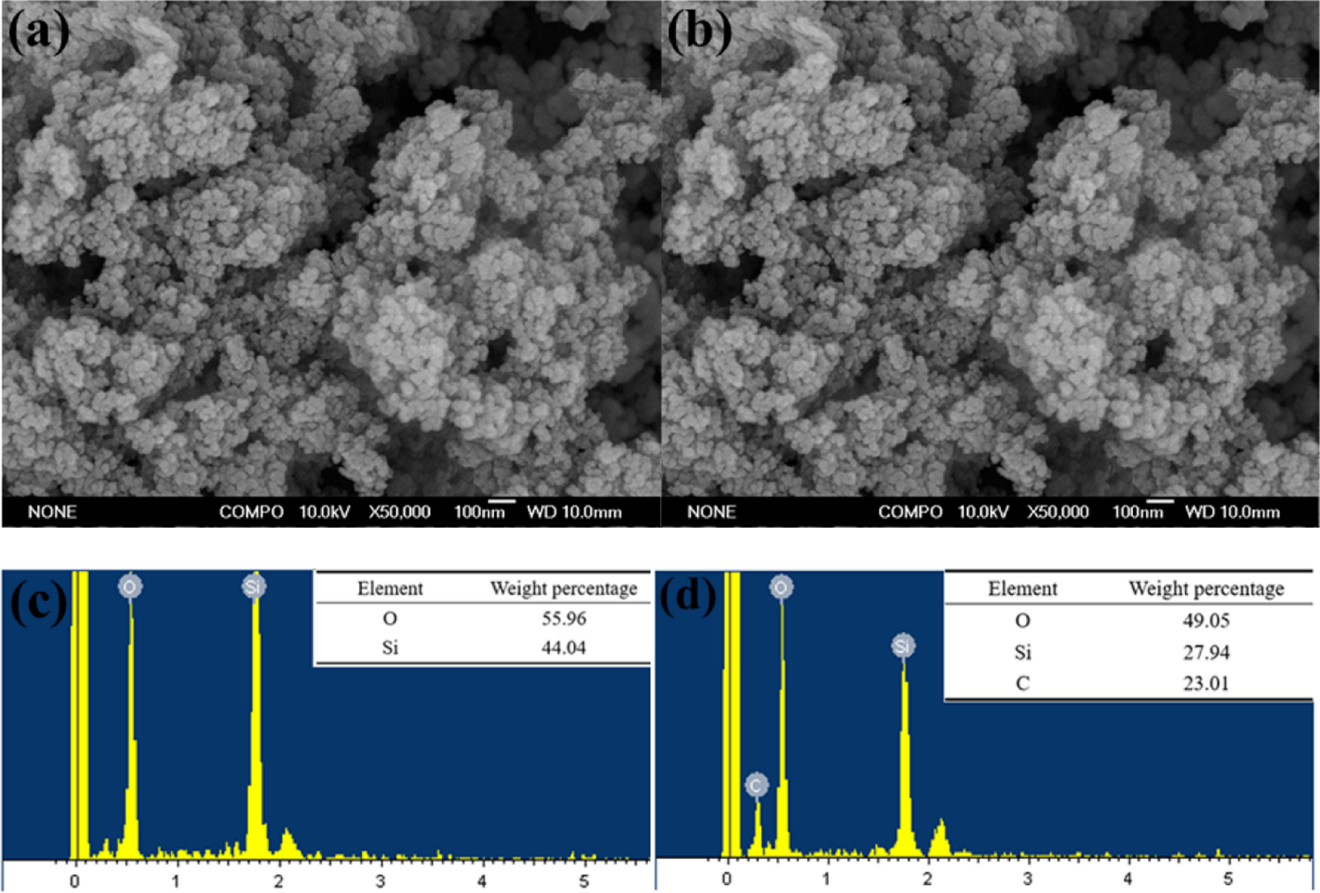
SEM images of nano-SiO_2_ (a) and HDTMS-nano-SiO_2_ (b). EDS images of nano-SiO_2_ (c) and HDTMS-nano-SiO_2_ (d).

The SEM images are shown in Figure 6a,b. It could be found that the particle size of nano-SiO_2_ before and after modification was relatively uniform, which was about tens of nanometers. The agglomeration phenomenon appeared, but the agglomeration degree of HDTMS-nano-SiO_2_ was slightly lower than that of nano-SiO_2_ because of the reduction of hydroxyl groups on the surface after modification. In the process of practical application, it can be dispersed in the corresponding solvent by means of mechanical stirring and ultrasonic wave. In addition, the chemical element composition of nano-SiO_2_ and HDTMS-nano-SiO_2_ were measured, which is demonstrated in Figure 6c,d. According to the comparative analysis of the data in the figures, it is apparent that the elemental composition of nano-SiO_2_ was only Si and O. After modification, a certain proportion of the C element was added to the elemental composition of HDTMS-nano-SiO_2_, which confirmed the modification of nano-SiO_2_. The presence of −CH_2_– and −CH_3_ led to the appearance of the C element.

## 3 Conclusions

In this study, nano-SiO_2_ with a ratio of 0.25:1 to HDTMS was utilized to modify nano-SiO_2_. Consequently, hydrophobic HDTMS-nano-SiO_2_ was obtained, the WCA of which reached 170.9°. Through FTIR and 2D-COS analyses, it was verified that nano-SiO_2_ had been successfully modified and that the sequence of the reaction of functional groups in the modification reaction was −CH_3_, −CH_2_–, and hydroxyl groups. The results of TG analysis showed that HDTMS-nano-SiO_2_ was weightless in a temperature range of 150–500 °C due to the presence of long-chain alkyl. By observing the SEM images, it could be found that HDTMS-nano-SiO_2_ agglomerated less because of the reduction of the hydroxyl groups. EDS elemental analysis also further confirmed the existence of the C element in HDTMS-nano-SiO_2_. This research can provide a reference for the research for the hydrophobic modification of nano-SiO_2_.

## 4 Experimental Section

### 4.1 Reagents

Nano-SiO_2_ with an average particle size of tens of nanometers and HDTMS (≥85%) were purchased from McLean Biochemical Technology Co., Ltd., (Shanghai, China). Anhydrous ethanol (≥99.7%) was purchased from Modern Oriental Science and Technology Development Co., Ltd., (Beijing, China). All of the above reagents were analytically pure and had not been further purified before use.

### 4.2 Preparation of HDTMS-Nano-SiO_2_

When the ratio of nano-SiO_2_ and HDTMS was 1.4, HDTMS-nano-SiO_2_ could be superhydrophobic. After being blended with organic silane, it could be made into composite coatings. These coatings showed hydrophobicity on paper, glass, metals, and other materials, with all of the WCA values reached 150°.^11^ According to the previous research, nano-SiO_2_ and HDTMS with different mass ratios of 0.25:1, 0.5:1, 1:1, 1:0.5, and 1:0.25 were prepared. They were dispersed in 300 mL of anhydrous ethanol and stirred for 1 h in a thermostatic water bath at 90 °C, separately. Then, the obtained products were cleaned with deionized water and filtered, and the solid part was dried in a vacuum drying oven at 60 °C for 24 h. Finally, HDTMS-nano-SiO_2_ was obtained.

### 4.3 Characterization

- WCA: Static water contact angles before and after modification of nano-SiO_2_ were measured by a contact angle analyzer (OCA-20, Germany). Deionized water (2 μL) was used as the test fluid. Five parts of each sample were measured and the mean value was calculated to characterize and analyze the change in its hydrophobic performance.
- FTIR: FTIR (Spectrum GX) was carried out using the potassium bromide tablet method. The spectral range was 400–4000 cm^–1^, and each sample was scanned 32 times with a resolution of 4 cm^–1^. The sample powder was mixed with the potassium bromide powder in a ratio of 1:100, then the sampler–powder mixture was ground with a mortar and pestle to ensure uniformity, and the tablet was pressed at a pressure of 10 Mpa.
- 2D-COS: The samples were loaded into the variable temperature accessories and heated at a heating rate of 2 °C/min. The infrared spectra were collected every 10 °C with a temperature range of 50–120 °C. The 2D analysis software 2DShige developed by Shigeaki Morita was used for data analysis and the 2D infrared spectra were obtained.^45−47^
- TG: TG analysis was conducted by a thermogravimetric analyzer (TA Q50) in the atmosphere of nitrogen, which was meant to test and investigate the thermal stability of nano-SiO_2_ before and after modification. The temperature range was 20–600 °C, and the heating rate was 10 °C/min in the process.
- SEM: SEM (JSM-6700F, Japan) was utilized to characterize and analyze the microscopic morphology of nano-SiO_2_ before and after modification, and the samples were pasted with a conductive adhesive and sprayed with gold for observation. In addition, the elements of the sample were tested quantitatively by instrument-prepared energy-dispersive X-ray spectroscopy (EDS).
